# Exceptional response of skin symptoms to secukinumab treatment in a patient with SAPHO syndrome: Case report and literature review

**DOI:** 10.1097/MD.0000000000030065

**Published:** 2022-08-19

**Authors:** Qiang Ji, Qing Wang, Wenping Pan, Yanfeng Hou, Xiuhua Wang, Lin Bian, Zhankui Wang

**Affiliations:** a First Clinical Medical College, Shandong University of Traditional Chinese Medicine, Jinan, Shandong, China; b Department of Rheumatology and Autoimmunology, Shandong Provincial Qianfoshan Hospital, First Clinical Medical College, Shandong University of Traditional Chinese Medicine, Shandong Key Laboratory of Rheumatic Disease and Translational medicine, Shandong medicine and Health Key Laboratory of Rheumatism; c Department of Rheumatology and Autoimmunology, The First Affiliated Hospital of Shandong First Medical University & Shandong Provincial Qianfoshan Hospital, Shandong Key Laboratory of Rheumatic Disease and Translational medicine, Shandong medicine and Health Key Laboratory of Rheumatism.

**Keywords:** palmoplantar pustulosis, SAPHO syndromesecukinumab, skin disorder

## Abstract

**Rationale:**

SAPHO syndrome is a rare clinical entity characterized by a wide range of dermatological and musculoskeletal manifestations. Treatment strategies are not standardized. Palmoplantar pustulosis (PPP) is the most common rash in patients with SAPHO syndrome.

**Patient concerns:**

A 24-year-old Chinese woman with no relevant medical or familial history had a 1-year history of cutaneous lesions with PPP and pain in the sternoclavicular joint.

**Diagnosis:**

Based on the diagnostic criteria for SAPHO syndrome proposed by Nguyen et al in 2012, we diagnosed SAPHO syndrome with severe PPP as the predominant manifestation.

**Interventions:**

Due to the limited therapeutic efficacy of methotrexate and cyclosporin, we started therapy with subcutaneous secukinumab 150 mg weekly for the first month, then 150 mg monthly thereafter.

**Outcomes:**

After 4 weeks of secukinumab administration, the patient showed significant remission of pustular skin lesions, with almost no joint pain and no adverse reaction. Complete remission of skin symptoms was achieved after 3 months. Joint pain and adverse events have not reoccurred in follow-up thus far.

**Conclusions:**

In patients with SAPHO syndrome, we recommend personalized treatment, which may have excellent therapeutic efficacy in those with PPP or severe skin symptoms. Although data related to the use of IL-17 blockers for SAPHO syndrome are very limited, secukinumab provides a novel therapeutic option, especially for patients with PPP and severe skin lesions. Further prospective studies are needed to support our findings.

## 1. Introduction

SAPHO syndrome^[1]^ is a rare clinical entity characterized by a wide range of dermatological and musculoskeletal manifestations. Palmoplantar pustulosis (PPP) is a chronic, inflammatory, and recurrent skin disease in the psoriasis spectrum.^[2]^ It is characterized by an eruption of sterile pustules on the palms and soles and is the most common type of rash in patients with SAPHO syndrome.^[1]^ Secukinumab is a human monoclonal antibody that targets IL-17A and is proven to be effective in the treatment of autoimmune psoriasis, psoriatic arthritis, and ankylosing spondylitis. Limited data are available in the literature on the use of secukinumab in SAPHO syndrome due to the rarity of treatment. We present a patient with SAPHO syndrome with severe PPP who had a dramatic response to secukinumab treatment and near-complete recovery after 3 months. We describe the follow-up of patients with SAPHO syndrome treated with secukinumab. Because of the limited pertinent data, we reviewed all previous studies involving patients with SAPHO syndrome treated with secukinumab. Essentially, this represents a permanent open cohort of patients, with ongoing inclusion of patients with SAPHO syndrome treated with secukinumab.

## 2. Patient information and Clinical findings

**Figure 1.**
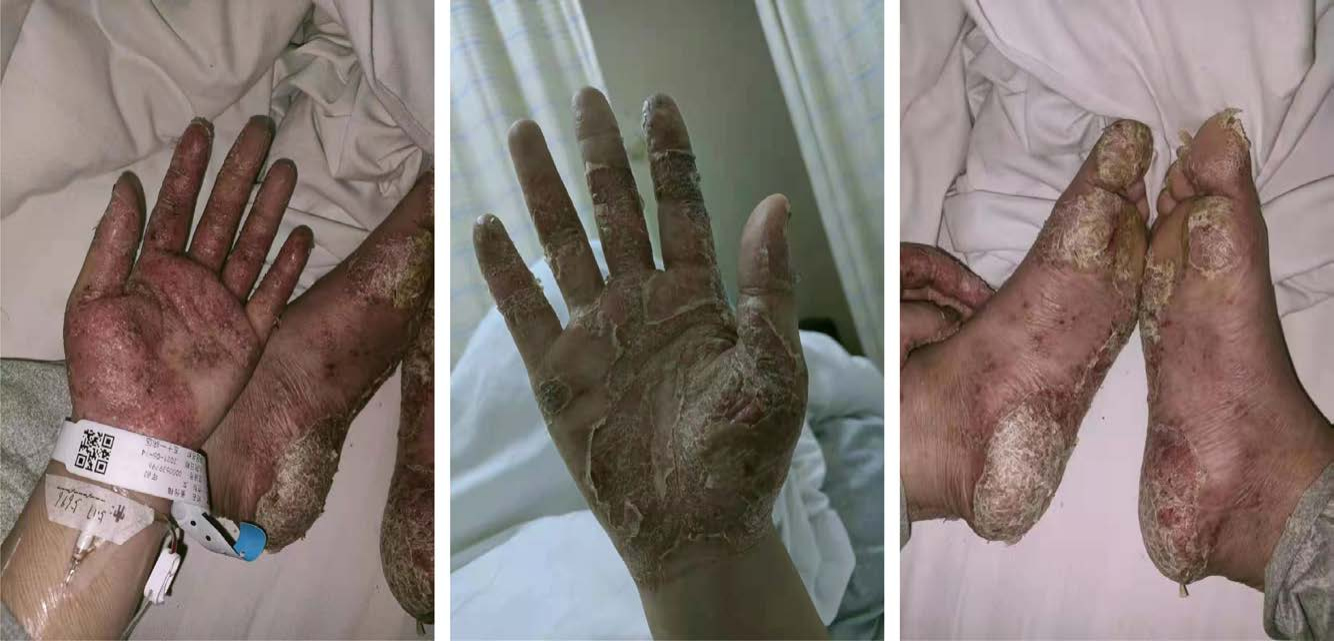
Pustular skin lesions distributed largely on the patient’s palms bilaterally and plantar feet at the initial visit before treatment.

A 24-year-old Chinese woman presented with a chief complaint of severe rash, flaking skin, itching and tenderness of the skin, pus ulceration, and pain in the sternoclavicular joint, along with a 1-year history of SAPHO syndrome with PPP. In the past year, the skin lesions had spread from the bilateral palms and soles to the limbs and scalp. Prior pharmacological treatment included methotrexate, cyclosporine, tretinoin, and calcipotriol ointment, with almost no improvement in symptoms. Physical examination revealed numerous pustular skin lesions on the palms bilaterally, plantar feet, and lower extremities, with partial ulceration, desquamation, and bilateral sternoclavicular joint tenderness (Fig. 1). Based on the diagnostic criteria proposed by Nguyen et al.^[3]^ in 2012 in *Semin Arthritis Rheum*, we diagnosed SAPHO syndrome with severe PPP as the predominant manifestation.

### 2.1. Timeline

**Figure 2.**
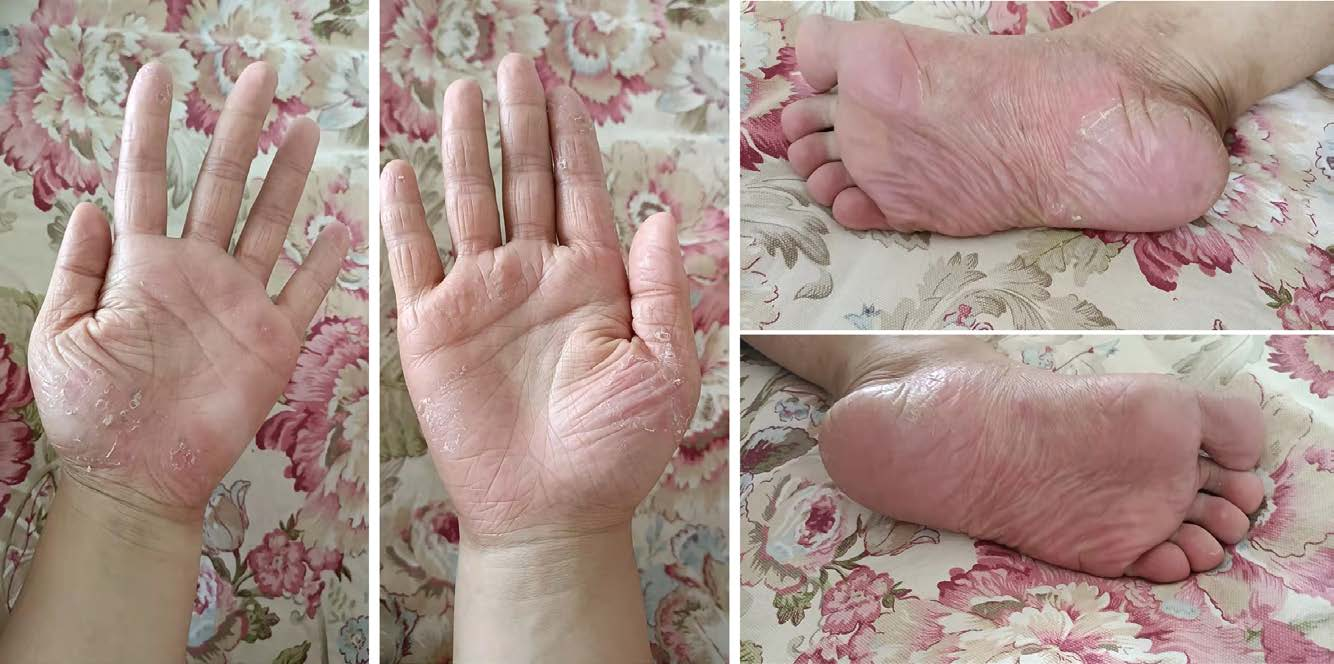
Bilateral palms show only partial desquamation after secukinumab therapy for 4 weeks. Near resolution of palmoplantar pustulosis in plantar feet bilaterally, after secukinumab therapy for 4 weeks.

The patient was admitted on May 14,2021. Her symptoms were rated using a series of scales: PASI, 22.4; BSA, 18%; DLQI, 28; PGA, 4; and VAS, 6. Bone scintigraphy revealed increased uptake in the left first rib. Blood test results were unremarkable, with no autoantibodies and normal acute-phase reactants. Due to the limited therapeutic efficacy of methotrexate, cyclosporine, and other drugs, we started therapy with subcutaneous secukinumab (Novartis Pharma Stein AG) 150 mg weekly for the first month and monthly thereafter. Four weeks later, the patient achieved significant remission of pustular skin lesions (Fig. 2), with almost no joint pain and no adverse reaction (PASI, 1.2; BSA, ≤1%; DLQI, 1; PGA, 1; VAS, 1).

**Figure 3.**
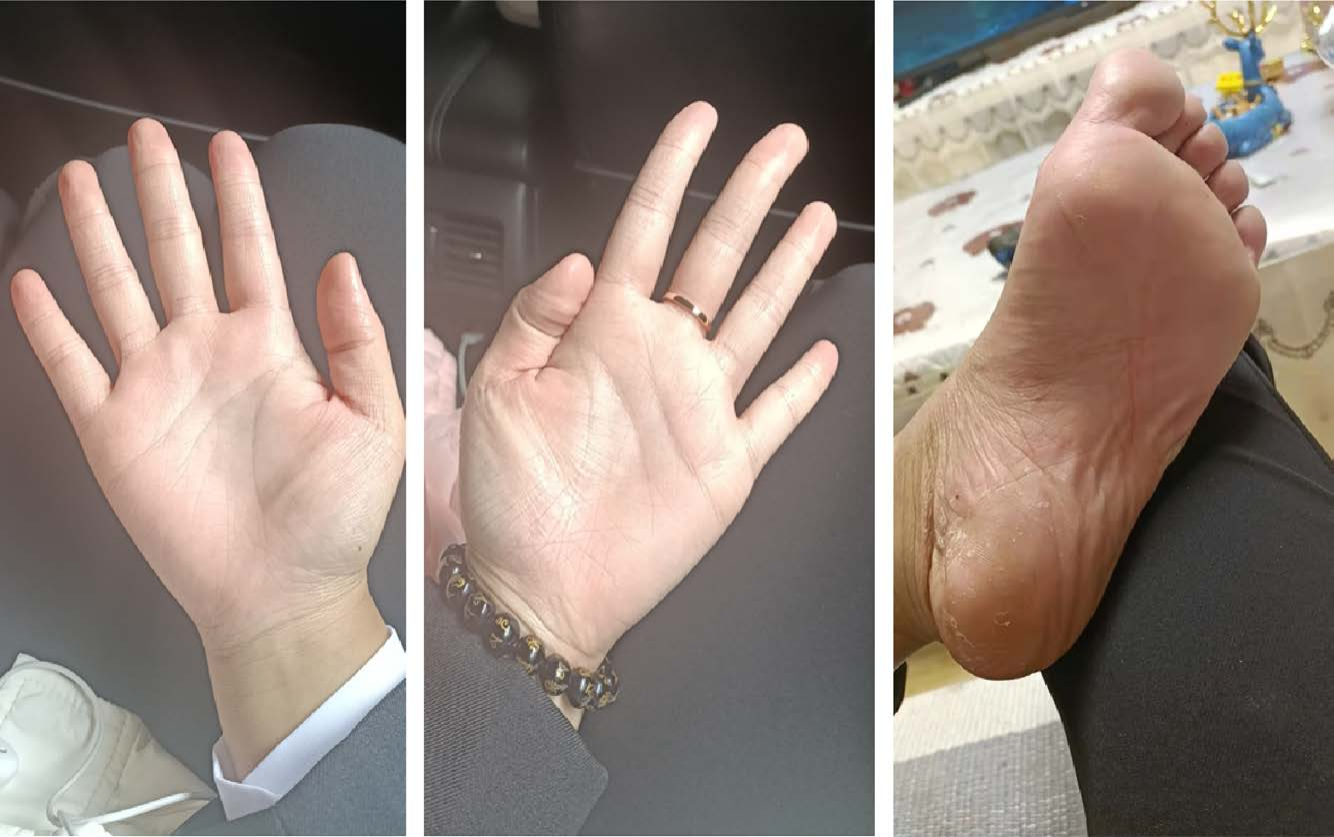
Completely resolution of palmoplantar pustulosis after 3 months of secukinumab therapy.

**Figure 4.**
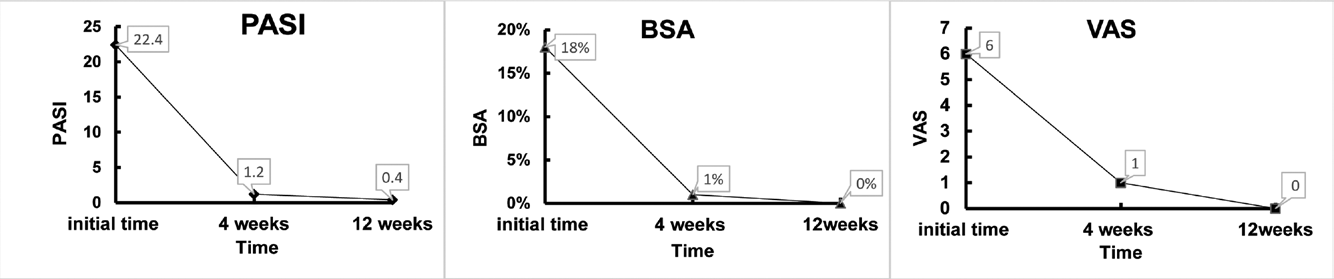
Clinical assessment of osteoarticular pain and skin lesion at the first visit, 4 weeks and 12 weeks. BSA = body surface area; PASI = Psoriasis Area Severity Index; VAS = visual analog scale for osteoarticular pain.

The patient achieved complete remission of skin symptoms (Fig. 3) after 3 months of follow-up, with no joint pain or adverse events (PASI, 0.4; BSA, 0%; DLQI, 1; PGA, 1; VAS, 0). The scores of PASI, BSA, and VAS at the first visit, 4 weeks, and 12 weeks are shown in Figure 4.

The patient returned to the hospital every month to report any adverse reactions. The most recent follow-up was on December 30,2021, and her symptoms had resolved completely without complications or adverse reactions.

## 3. Literature search

**Figure 5.**
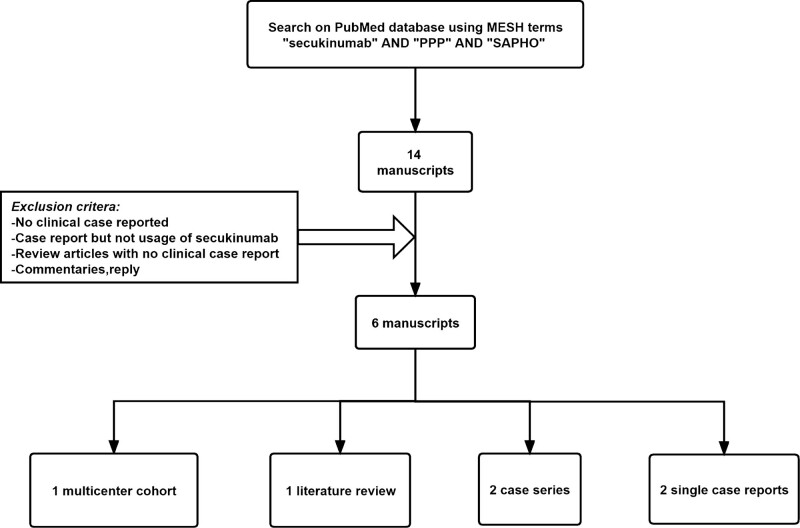
Relevant literature review methodology flowchart.

We performed a systematic electronic literature search on PubMed using the key words “secukinumab” AND “SAPHO”, up to January 20, 2022, without other restrictions on publication date. The search yielded 8 articles. The abstracts of these articles were evaluated to identify studies on the therapeutic use of secukinumab in patients with SAPHO syndrome. Only 6 articles met the search criteria and were included in the analysis (1 multicenter cohort, 1 literature review containing cases, 2 case series, and 2 single case reports), as illustrated in the flow chart (Fig. 5). Table 1 presents the characteristics of the 10 cases.

**Table 1:** Demographics, clinical features, previous treatments, and outcomes after secukinumab treatment in patients with SAPHO syndrome.

#	Reference/publication (yr)	Age (yr)	Sex	Disease duration (yr)	Osteoarticular manifestations	Skin manifestations	Previous treatments	Response of osteoarticular	Response of skin manifestations	Adverse event
1	Nikolakis G et al/2021	50	F	13	Knee joints arthritis, wrist joints arthritis, and synovitis	HS, AC, PG, PPP	Isotretinoin, ADA	CR	SR	None
2	Boyuan Sun et al/2021	31	M	9	Left jaw pain and mouth opening limitation	Unknown	Pamidronate, tofacitinib, ADA	SR	Unknown	None
3	Dimitrios Daoussis et al/2019	53	F	7	Bilateral shoulder arthritis, osteitis of the sternum and both clavicles, hyperostosis at the sternoclavicular joints	PPP	Zoledronic acid, MTX, INF, ADA	CR	CR	None
4	Daniel Wendling et al/2017	37	F	3	Axial spondyloarthritis, ACW involvement, peripheral arthritis	PPP	MTX, ADA, INF, UST	NR	SR	None
5		64	M	5	Axial spondyloarthritis, ACW involvement	PPP	MTX, SZP, INF, ADA	NR	PR	None
6		46	F	5	ACW involvement, spondyloarthritis, arthritis	PPP	MTX, SASP, INF, ANAK	NR	NR	Paradoxical psoriasis
7	Lun Wang et al/2021	30	F	0.7	ACW, spine, sacroiliac region, and shoulder involvement	PPP, nail lesions	NSAID	CR	PPP, SR; Nail lesions, aggravation	Otitis media, tonsillitis
8		51	M	1	ACW, spine, sacroiliac region, shoulder, and hip involvement	PPP, PV, nail lesions	NSAID	SR	PPP, SR; PV, SR; Nail lesions, stable	None
9		49	F	12	ACW, spine, and shoulder involvement	PPP, PV, nail lesions	TNFi, THD, SASP, NSAID	SR	PPP, SR; PV, SR; Nail lesions, aggravation	Herpes zoster
10		42	F	4	ACW, spine, sacroiliac region, and shoulder involvement	PPP, PV, nail lesions	NSAID	SR	PPP, SR; PV, CR; Nail lesions, aggravation	Fungal external otitis, dyslipidemia

AC = acne conglobate, ACW = anterior chest wall, ADA = adalimumab, ANAK = anakinra, CR = complete resolution, HS = hidradenitis suppurativa, INF = infliximab, MTX = methotrexate, NR = no remission, PG = pyoderma gangrenosum, PPP = palmoplantar pustulosis, PR = partial remission, PV = psoriasis vulgaris, SASP = salicylazosulfapyridine, SR = significant remission, SZP = salazosulfapyridine, TNFi = tumor necrosis factor inhibitor, UST = ustekinumab.

### 3.1. Case reports

Nikolakis et al.^[4]^ described 1 patient treated with secukinumab 300 mg subcutaneously weekly for the first month and then monthly for 4 months. A 50-year-old woman presented with confluent erythematous pustules on the palms and soles with psoriasis-like scaling of the lesions, followed by intermittent pain and swelling of the shoulders and knees. The pattern of the bull’s head sign^[5]^ was detected using a bone scan. Secukinumab treatment was effective, leading to significant remission of joint pain and pustular psoriasis after 4 months.

Sun et al.^[6]^ reported 1 patient treated with secukinumab (No secukinumab administration details were reported). A 31-year-old man with a 9-year history of SAPHO syndrome presented with significant left jaw pain and limited mouth opening. The patient was successively treated with pamidronate, tofacitinib, and adalimumab; however, none of the treatments achieved long-term remission. Subsequently, secukinumab was administered to the patient. Magnetic resonance imaging of the maxillofacial region revealed remarkable remission of edema in the left masseter and mandibular periosteum after 2 months of treatment. His jaw pain, mouth-opening limitation, and inflammatory indicators also improved significantly, and no recurrence was observed.

Daoussis et al.^[7]^ described 1 patient treated with secukinumab 300 mg subcutaneously weekly for the first month and then monthly for 9 months. The patient was a 53-year-old woman who presented with SAPHO syndrome characterized by discomfort and pain in the upper anterior chest wall and both shoulders and erythematous/pustular lesions on the palms and soles. After repeated illness due to the use of zoledronic acid, methotrexate, infliximab, and adalimumab, secukinumab was used for treatment and the pain subsided significantly after 2 weeks. After 9 months of treatment, the patient remained symptom free with no side effects related to treatment. It is noteworthy that this case provides the first radiographic evidence of the effective suppression of osteitis by IL-17 blockade in SAPHO syndrome.

Wendling et al.^[8]^ reported the results of 3 courses of secukinumab therapy in patients with SAPHO syndrome unresponsive to previous treatments. The first patient was a 37-year-old woman with anterior chest wall and axial involvement, peripheral arthritis, and PPP with secukinumab 150 mg subcutaneously weekly for the first month, then monthly for 3 months. There was an improvement in skin lesions but no major improvement in skeletal involvement after 3 months of secukinumab treatment. The second patient, a 64-year-old man, had PPP, anterior chest wall and axial involvement, and arthritis was treated with secukinumab 300 mg subcutaneously weekly for the first month and then monthly for 3 months. Four weeks after the initiation of secukinumab, there was improvement in his skin disease, but musculoskeletal symptoms did not improve. The third patient was a 46-year-old woman with anterior chest wall involvement, spondyloarthritis, arthritis, and PPP treated with secukinumab 150 mg subcutaneously weekly for the first month and then monthly for 3 months. Unfortunately, the patient skin and musculoskeletal symptoms did not improve, and paradoxical psoriasis occurred.

Wang et al.^[9]^ reported a case series of 4 patients with SAPHO syndrome treated with 24-week secukinumab (150 mg subcutaneous once weekly for 4 weeks and every 4 weeks thereafter). The first patient was a 30-year-old woman with involvement of the anterior chest wall, spine, sacroiliac region, and shoulder, PPP, and nail lesions. Secukinumab treatment was highly effective in resolving osteoarticular pain and PPP; however, the nail disease was aggravated and otitis media and tonsillitis occurred. The second patient was a 51-year-old man with anterior chest wall, spine, sacroiliac region, shoulder, and hip involvement, PPP, psoriasis vulgaris, and nail lesions. The effect of secukinumab was pronounced and rapid, with complete elimination of all symptoms except nail lesions, and no adverse events occurred. The third patient was a 49-year-old woman with anterior chest wall, spine, and shoulder involvement, PPP, psoriasis vulgaris, and nail lesions. The patient exhibited rapid improvement of skin lesions and osteoarticular pain, although herpes zoster infection occurred. The fourth patient was a 42-year-old woman with involvement of the anterior chest wall, spine, sacroiliac region, and shoulder, PPP, psoriasis vulgaris, and nail lesions. Secukinumab was again effective, leading to complete remission of musculoskeletal manifestations and skin lesions; however, external fungal otitis and dyslipidemia occurred. No severe adverse events were reported. This is currently the largest case series on secukinumab treatment for SAPHO syndrome. This study showed that secukinumab has the potential to induce complete remission of both osteoarticular and cutaneous lesions in patients with SAPHO syndrome.

### 3.2. A multicenter cohort study

Mrowietz et al.^[10]^ conducted a phase 3b multicenter, randomized, double-blind, placebo-controlled, parallel group study comparing 52-week treatments with 300 mg of secukinumab (n = 79), 150 mg of secukinumab (n = 80), and placebo (n = 78) in patients with moderate-to-severe PPP. The results were encouraging: At week 52, a PPPASI75 response was achieved in 41.8% of subjects treated with 300 mg of secukinumab (33 of 79) compared to 35.0% of subjects treated with 150 mg of secukinumab (28 of 80), and no unexpected adverse events were observed. The exceptional efficacy of secukinumab in PPP treatment was fully illustrated in this study.

## 4. Discussion

The incidence of PPP is estimated to range from 0.01 to 0.05%.^[11]^ In severe cases, some lesions gradually enlarge and finally merge, forming large erythematous-squamous patches covering the entire palmar and plantar surfaces, sometimes with fissure formation and excruciating pain,^[12]^ which appeared in the cases we introduced.

SAPHO syndrome^[1]^ encompasses a special group of symptoms, including synovitis, acne, pustulosis, hyperostosis, and osteitis, and was first diagnosed by Chamot et al in 1987. It most commonly presents with acne or PPP. It is an intractable inflammatory disease that causes skin rash, sternoclavicular/sacroiliac/peripheral arthritis, and enthesitis.^[3]^ Similar to ankylosing spondylitis, SAPHO syndrome may also be considered a spondyloarthropathy^[13]^; however, there is no clear agreement in this respect. In recent years, biologics have been increasingly used for the treatment of SAPHO syndrome, with TNF-α antagonists being the most used. Many case reports and a case series have shown that TNF-α antagonists can significantly improve skin lesions and bone pain symptoms in patients with SAPHO syndrome.^[14,15]^ However, some studies^[16]^ have shown that, in patients with rheumatic immune disease treated with TNF-α antagonists, a small number of patients have new skin lesions or aggravated original skin lesions during treatment. Therefore, as a biological agent with strong advantages in the treatment of severe psoriasis,^[17]^ secukinumab may have good prospects in the treatment of SAPHO syndrome with severe PPP. The cytokine IL-17 has been highlighted in recent years since it was implicated in the pathophysiology of many rheumatic diseases.^[18]^ Interestingly, IL-17 is involved at least to some extent in SAPHO syndrome.^[7]^

Secukinumab is a fully human IgG1 anti-IL-17A monoclonal antibody that selectively suppresses the inflammatory cascade induced by IL-17A.^[19]^ Some phase III^[19–25]^ clinical trials have confirmed IL-17A as an important target for the treatment of psoriasis and psoriatic arthritis. In the treatment of psoriasis, secukinumab was found superior to several contemporary biological agents currently used, such as etanercept and ustekinumab.^[17]^ Etanercept^[26]^ is a tumor necrosis factor antagonist approved for the treatment of psoriatic arthritis and psoriasis. The antiinflammatory effects of etanercept are due to its ability to bind the proinflammatory cytokine, TNF, preventing it from interacting with cell-surface receptors. Ustekinumab^[27]^ is a fully human immunoglobulin G1k monoclonal antibody that specifically blocks the shared p40 subunit of IL-12 and IL-23, naturally occurring regulatory cytokines involved in inflammatory and immune responses, natural killer cell activation, and signaling for downstream effector cytokine production (e.g., TNF, IL-17, IL-22). Secukinumab is approved for the treatment of ankylosing spondylitis in several countries including the United States, China, and the European Union. Our choice of secukinumab over other biologics for the treatment of this patient was based on its excellent therapeutic effect in severe psoriasis and ankylosing spondylitis. Some studies^[28–30]^ have confirmed increased activity of IL-17A in PPP and have suggested that IL-17 may play a key role in inflammation in PPP. Activation of the T-helper 17 (Th17) axis has been found in patients with SAPHO syndrome with a prolonged course.^[31]^ There may be a rationale for blocking IL-17 in SAPHO syndrome, and this might allow better disease control. Although there are limited data on the efficacy of secukinumab in the treatment of SAPHO, 1 may speculate that secukinumab can also treat SAPHO syndrome due to the relationship between secukinumab, SAPHO, PPP, and IL-17. Our report documents a successful secukinumab treatment of SAPHO syndrome with severe PPP in a patient who was not treated with immunosuppressants.

Seven of the 10 patients with SAPHO syndrome treated with secukinumab in the literature experienced significant therapeutic effects. Only 1 patient showed no improvement in skin symptoms, and 3 patients showed no improvement in bone and joint symptoms. Coincidentally, due to the extremely severe skin damage in our patient, we used secukinumab instead of other biologics. Two patients reported by Wang et al.^[9]^ presented findings similar to those of our patient, and both had the same shorter disease course. No severe adverse events occurred in any of the patients, including those whom we searched for and reported. We should note that our patient, similar to those reported by Wang et al, achieved excellent results using only 150 mg of subcutaneous secukinumab, which may be related to Asian ethnicity.

The clinical manifestations of SAPHO syndrome include symptoms of the skin, bones, and joints. Skin symptoms can appear earlier or later than bone and joint symptoms, and can also appear simultaneously. Our assessment of severity of these symptoms was biased. Due to the lack of large number of prospective controlled studies and the atypical early symptoms of the disease, there is no unified guideline or consensus on the current treatment strategy. We suggest individualized treatment for patients with SAPHO syndrome. For example, our patient had more severe skin symptoms than bone and joint symptoms when admitted to hospital; therefore, we chose to use secukinumab, which has an obvious therapeutic effect on moderate-to-severe PPS and ankylosing spondylitis, thus achieving an excellent curative effect.

## 5. Conclusion

SAPHO syndrome is a rare disease, and its treatment remains empirical. We recommend individualized treatment for patients with SAPHO syndrome. Secukinumab may be a better choice when skin symptoms are severe. Although data related to the use of IL-17 blockers in SAPHO syndrome are very limited, secukinumab provides a novel therapeutic option, especially for patients with PPP and severe skin lesions. If the skin symptoms were less severe than bone and joint symptoms, we would still recommend the use of secukinumab, based on the fact that there are many cases in which secukinumab has improved bone and joint symptoms in the literature we reviewed, and also based on the efficacy of secukinumab in ankylosing spondylitis that has long been recognized. And a permanent open-label patient cohort has been developed in our department to continuously enroll patients with SAPHO syndrome (regardless of whether skin symptoms are more severe than bone and joint symptoms) receiving secukinumab. Further prospective studies are needed to support our findings.

## Author contributions

Conceptualization: Zhankui Wang.

Data curation: Qing Wang.

Investigation: Xiuhua Wang, Lin Bian.

Resources: Qing Wang.

Supervision: Wenping Pan, Yanfeng Hou.

Writing - original draft: Qiang Ji.

Writing - review & editing: Qiang Ji, Zhankui Wang.

## Acknowledgments

The authors thank the participating patients, as well as Editage (www.editage.com) for English language editing.

## References

[1] Chamot AM Benhamou CL Kahn MF. Acne-pustulosis-hyperostosis-osteitis syndrome. Results of a national survey. 85 cases. Rev Rhum Mal Osteoartic. 1987;54:187–96. 2954204

[2] Misiak-Galazka M Zozula J Rudnicka L. palmoplantar pustulosis: recent advances in etiopathogenesis and emerging treatments. Am J Clin Dermatol. 2020;21:355–70. 32008176 10.1007/s40257-020-00503-5 PMC7275027

[3] Nguyen MT Borchers A Selmi C. The SAPHO syndrome. Semin Arthritis Rheum. 2012;42:254–65. 23153960 10.1016/j.semarthrit.2012.05.006

[4] Nikolakis G Kreibich K Vaiopoulos A. Case report: PsAPSASH syndrome: an alternative phenotype of syndromic hidradenitis suppurativa treated with the IL-17A inhibitor secukinumab. F1000Res. 2021;10:381. 34540202 10.12688/f1000research.52100.1 PMC8424462

[5] Kundu BK Naik AK Bhargava S. Diagnosing the SAPHO syndrome: a report of three cases and review of literature. Clin Rheumatol. 2013;32:1237–43. 23604547 10.1007/s10067-013-2251-1

[6] Sun B Cao Y Wang L. Successful treatment of refractory mandibular lesions in SAPHO syndrome with secukinumab. Rheumatology (Oxford). 2021;60:473–4. 32712673 10.1093/rheumatology/keaa352

[7] Daoussis D Konstantopoulou G Kraniotis P. Biologics in SAPHO syndrome: a systematic review. Semin Arthritis Rheum. 2019;48:618–25. 29773231 10.1016/j.semarthrit.2018.04.003

[8] Wendling D Aubin F Verhoeven F. IL-23/Th17 targeted therapies in SAPHO syndrome. a case series. Joint Bone Spine. 2017;84:733–5. 28532819 10.1016/j.jbspin.2017.05.016

[9] Wang L Sun B Li C. Clinical and radiological remission of osteoarticular and cutaneous lesions in sapho patients treated with secukinumab: a case series. J Rheumatol. 2021;48:953–5. 33649072 10.3899/jrheum.201260

[10] Mrowietz U Bachelez H Burden AD. Secukinumab for moderate-to-severe palmoplantar pustular psoriasis: results of the 2PRECISE study. J Am Acad Dermatol. 2019;80:1344–52. 30716404 10.1016/j.jaad.2019.01.066

[11] Mrowietz U van de Kerkhof PC. Management of palmoplantar pustulosis: do we need to change? Br J Dermatol. 2011;164:942–6. 21275942 10.1111/j.1365-2133.2011.10233.x

[12] Murakami M Terui T. Palmoplantar pustulosis: current understanding of disease definition and pathomechanism. J Dermatol Sci. 2020;98:13–9. 32201085 10.1016/j.jdermsci.2020.03.003

[13] Dougados M van der Linden S Juhlin R. The European spondylarthropathy study group preliminary criteria for the classification of spondylarthropathy. Arthritis Rheum. 1991;34:1218–27. 1930310 10.1002/art.1780341003

[14] Firinu D Murgia G Lorrai MM. Biological treatments for SAPHO syndrome: an update. Inflamm Allergy Drug Targets. 2014;13:199–205. 24846337 10.2174/1871528113666140520100402

[15] Hampton SL Youssef H. Successful treatment of resistant SAPHO syndrome with anti-TNF therapy. BMJ Case Rep. 2013. 10.1136/bcr-2012-007161 PMC3603525 23355559

[16] Lee HH Song IH Friedrich M. Cutaneous side-effects in patients with rheumatic diseases during application of tumour necrosis factor-alpha antagonists. Br J Dermatol. 2007;156:486–91. 17300238 10.1111/j.1365-2133.2007.07682.x

[17] Abrouk M Gandy J Nakamura M. Secukinumab in the treatment of psoriasis and psoriatic arthritis: a review of the literature. Skin Therapy Lett. 2017;22:1–6. 28732152

[18] Miossec P. Update on interleukin-17: a role in the pathogenesis of inflammatory arthritis and implication for clinical practice. RMD Open. 2017;3:e000284. 28243466 10.1136/rmdopen-2016-000284 PMC5318575

[19] Paul C Reich K Gottlieb AB. Secukinumab improves hand, foot and nail lesions in moderate-to-severe plaque psoriasis: subanalysis of a randomized, double-blind, placebo-controlled, regimen-finding phase 2 trial. J Eur Acad Dermatol Venereol. 2014;28:1670–5. 24393602 10.1111/jdv.12359

[20] Mease PJ McInnes IB Kirkham B. Secukinumab inhibition of interleukin-17a in patients with psoriatic arthritis. N Engl J Med. 2015;373:1329–39. 26422723 10.1056/NEJMoa1412679

[21] McInnes IB Sieper J Braun J. Efficacy and safety of secukinumab, a fully human anti-interleukin-17A monoclonal antibody, in patients with moderate-to-severe psoriatic arthritis: a 24-week, randomised, double-blind, placebo-controlled, phase II proof-of-concept trial. Ann Rheum Dis. 2014;73:349–56. 23361084 10.1136/annrheumdis-2012-202646

[22] McInnes IB Mease PJ Kirkham B. Secukinumab, a human anti-interleukin-17A monoclonal antibody, in patients with psoriatic arthritis (FUTURE 2): a randomised, double-blind, placebo-controlled, phase 3 trial. Lancet. 2015;386:1137–46. 26135703 10.1016/S0140-6736(15)61134-5

[23] Mrowietz U Leonardi CL Girolomoni G. Secukinumab retreatment-as-needed versus fixed-interval maintenance regimen for moderate to severe plaque psoriasis: a randomized, double-blind, noninferiority trial (SCULPTURE). J Am Acad Dermatol. 2015;73:27–36.e1. 25982539 10.1016/j.jaad.2015.04.011

[24] Thaçi D Blauvelt A Reich K. Secukinumab is superior to ustekinumab in clearing skin of subjects with moderate to severe plaque psoriasis: CLEAR, a randomized controlled trial. J Am Acad Dermatol. 2015;73:400–9. 26092291 10.1016/j.jaad.2015.05.013

[25] Blauvelt A Prinz JC Gottlieb AB. Secukinumab administration by pre-filled syringe: efficacy, safety and usability results from a randomized controlled trial in psoriasis (FEATURE). Br J Dermatol. 2015;172:484–93. 25132411 10.1111/bjd.13348

[26] Onsun N Güneş B Yabaci A. Retention and survival rate of etanercept in psoriasis over 15 years and patient outcomes during the COVID-19 pandemic: The real-world experience of a single center. Dermatol Ther. 2021;34:e14623. 33274541 10.1111/dth.14623 PMC7744860

[27] Ghosh S Gensler LS Yang Z. Ustekinumab safety in psoriasis, psoriatic arthritis, and Crohn’s disease: an integrated analysis of phase ii/iii clinical development programs. Drug Saf. 2019;42:751–68. 30739254 10.1007/s40264-019-00797-3 PMC6520311

[28] Hagforsen E Hedstrand H Nyberg F. Novel findings of Langerhans cells and interleukin-17 expression in relation to the acrosyringium and pustule in palmoplantar pustulosis. Br J Dermatol. 2010;163:572–9. 20426778 10.1111/j.1365-2133.2010.09819.x

[29] Bissonnette R Fuentes-Duculan J Mashiko S. Palmoplantar pustular psoriasis (PPPP) is characterized by activation of the IL-17A pathway. J Dermatol Sci. 2017;85:20–6. 27743912 10.1016/j.jdermsci.2016.09.019

[30] Bissonnette R Nigen S Langley RG. Increased expression of IL-17A and limited involvement of IL-23 in patients with palmo-plantar (PP) pustular psoriasis or PP pustulosis; results from a randomised controlled trial. J Eur Acad Dermatol Venereol. 2014;28:1298–305. 24112799 10.1111/jdv.12272

[31] Firinu D Barca MP Lorrai MM. TH17 cells are increased in the peripheral blood of patients with SAPHO syndrome. Autoimmunity. 2014;47:389–94. 24720503 10.3109/08916934.2014.906582

